# *Platonia insignis:* A Systematic Synthesis of Scientific Studies on Its Biology, Ecology, and Potential Applications

**DOI:** 10.3390/plants14060884

**Published:** 2025-03-12

**Authors:** Kira Figueredo Loiola Alves, Aldilene da Silva Lima, Priscila Marlys Sá Rivas, Irislene Cutrim Albuquerque, Jordanya Ferreira Pinheiro, Paulo Henrique Aragão Catunda, Sérgio Heitor Sousa Felipe, Fabrício de Oliveira Reis, Diego Silva Batista, Juliane Maciel Henschel, Fábio Afonso Mazzei Moura de Assis Figueiredo, Antônia Alice Costa Rodrigues, Thais Roseli Corrêa, Tiago Massi Ferraz

**Affiliations:** 1 Postgraduate Program in Agricultural Sciences, Tissue Culture Laboratory, State University of Maranhão, Av. Lourenço Vieira da Silva, São Cristóvão, São Luís 65055-310, MA, Brazil; kirafg8@gmail.com (K.F.L.A.); priscila.sarivas@gmail.com (P.M.S.R.); albuquerqueiris0@gmail.com (I.C.A.); jordanyaf.p@gmail.com (J.F.P.); sergio.h.s.felipe@gmail.com (S.H.S.F.); fareoli@gmail.com (F.d.O.R.); figueiredo.uema@gmail.com (F.A.M.M.d.A.F.); aacrodrigues@outlook.com (A.A.C.R.); thaiscorrea@professor.uema.br (T.R.C.); ferraztm@gmail.com (T.M.F.); 2 Center for Higher Studies of Coelho Neto, State University of Maranhão, Coelho Neto 65620-000, MA, Brazil; aldilene29@gmail.com; 3 Professional Master’s Program in National Network for Management and Regulation of Water Resources, State University of Maranhão, São Luís 65055-310, MA, Brazil; paulocatunda.uema@gmail.com; 4 Postgraduate Program in Agronomy, Federal University of Paraíba, Areia 58397-000, PB, Brazil; julianemhenschel@gmail.com

**Keywords:** amazon, fruit trees, *Platonia insignis*

## Abstract

*Platonia insignis*, a native tree from the Amazon, has a high market demand due to its various uses, such as producing pulps and ice creams from its fruit and furniture from its wood. This review aims to systematize the scientific knowledge about the species and explore the potential of biotechnology to elucidate its growth, development, and fruiting mechanisms. Databases such as the Web of Science, Scopus, the Brazilian Agricultural Research Database, and PubMed were consulted using keywords like “*P. insignis*”, “bacuri”, and “bacurizeiro”. Of the 67 selected articles, it was observed that rigorous research on *P. insignis* is limited. Current management is based on empirical observations, but biotechnology could expedite the domestication process. Additionally, studying medicinal compounds from *P. insignis* could open new economic opportunities, encourage germplasm conservation, and drive genetic breeding.

## 1. Introduction

*Platonia insignis* Mart. (Clusiaceae) is a tree species of considerable cultural, economic, environmental, and medicinal importance. It is primarily found in the Amazon biome, particularly in the Brazilian Amazon. Brazil accounts for approximately 86% of the fruit production of this species, with the state of Pará being a major contributor [1]. Research is concentrated in Brazilian states such as Amazonas, Pará, Maranhão, and Piauí [2,3,4,5]. These studies aim to mitigate limitations imposed by the species’ reproductive biology in extractive management contexts.

*Platonia insignis* is allogamous, requiring genotype diversity for fruiting, resulting in high genetic variability in progenies [6,7]. This poses challenges for domestication due to floral biology, and threats from agricultural expansion, illegal burnings, and urbanization further reduce its habitats [8]. Embrapa Amazônia Oriental and Embrapa Meio Norte have pioneered in situ and ex situ conservation strategies for *P. insignis* genetic resources [9,10].

Additionally, *P. insignis* has an extended seedling establishment period, often exceeding three years, while fruit maturation takes 10 to 12 years [11]. Family farmers and extractive communities have adopted empirical techniques, such as root sprouting, to shorten the juvenile phase and enhance fruit production. However, these practices lack robust scientific validation. Furthermore, information on effective postharvest conservation methods remains scarce [12].

Biotechnology, particularly the “omics”, can provide comprehensive insights into the genetic and molecular bases of desirable traits [13]. In vitro cultivation methods [14] can facilitate micropropagation, micrografting, somatic embryogenesis, and secondary metabolism research. This can advance the understanding of active principles and medicinal compounds in *P. insignis*.

Despite significant technical advancements in production, a deeper scientific understanding of *P. insignis* is still needed. Rigorous research incorporating robust statistical analyses and sample traceability can provide insights into the plant’s developmental stages and support the establishment of more effective cultivation, management, harvesting, and postharvest methods.

This study aimed to systematically review the scientific studies conducted on *P. insignis* across all fields of knowledge. This systematic review focuses on assessing the existing scientific literature on *P. insignis* across all areas of knowledge, based on the hypothesis that current research is insufficient to answer key questions about the species’ biology. This review will guide future basic and applied research and enhance knowledge about *P. insignis.*

## 2. Results and Discussion

**Figure 1:**
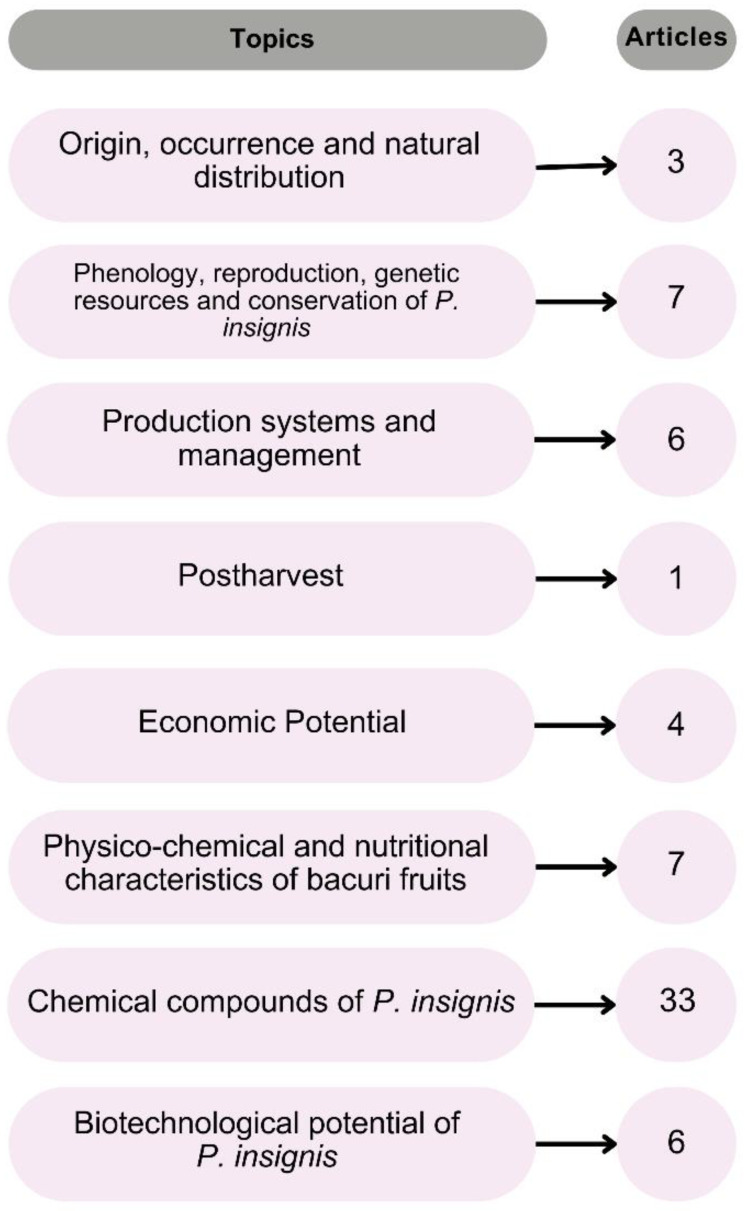
Article grouping and counts for results and discussion presentation.

A total of 67 articles were included in this systematic review and categorized into relevant content topics for the presentation and discussion of results, with occasional cross-references across topics. Following categorization, the topics and the corresponding number of articles were organized as shown in Figure 1.

### 2.1. Origin, Occurrence, and Natural Distribution

The origin of *P. insignis* is traced to the Brazilian Amazon [15]. While initial disagreements existed among researchers regarding its precise state of origin—whether Pará or Amazonas—all proposed locations (the Amazon River estuary, the right and left banks of the Pará River, and the southeastern Marajó Islands) lie within Pará. This suggests the species is most prevalent in the northeastern mesoregion of Pará state.

*Platonia insignis* trees are native to several Brazilian states. In the Brazilian Amazon, they are found in Acre, Amazonas, Roraima, and Tocantins, typically in primary forests with a very limited number of individuals per hectare. In northeastern Brazilian, *P. insignis* is present in Maranhão and Piauí, following the course of the Tocantins and Pará rivers and extending along the Gurupi River, both in primary and secondary forests. In South America, these trees are also observed in Peru, Colombia, Venezuela, and the Guyanas [7,15].

*Platonia insignis* trees thrive in transitional open vegetation with distinct rainy and dry seasons. Silva Ferreira et al. [16] assessed the photosynthetic plasticity of seven genotypes during the dry season and found that only one genotype responded well to environmental variations, maintaining good photosynthetic performance indices despite climatic changes. Generally, the occurrence and distribution environments of *P. insignis* are characterized by deep, weathered soils with low fertility and predominantly an Ami climate (a transition climate between Afi and Awi, with a dry season of two to three months and annual precipitation of 2000 mm or more) and an Awi climate (a clear dry season of five to six months and annual precipitation below 2000 mm), according to the Köppen classification [15,17].

### 2.2. Phenology, Reproduction, Genetic Resources, and Conservation of P. insignis

#### 2.2.1. Phenology and Reproduction of *P. insignis*

*Platonia insignis* trees are medium to large, ranging from 15 to 30 m in height and up to 1 m in trunk diameter. While some authors consider this species evergreen [7], others describe it as deciduous, with a leaf loss period of nearly 3 months starting in June or July [18]. The tree has large flowers (3 to 7 cm), which are pinkish-white to yellow, hermaphroditic, polystemonous, radially symmetrical, and exhibit diurnal anthesis [7]. Flower anthesis occurs after the full development of flower buds, which takes 11 to 21 days [18].

The flowers produce abundant nectar and pollen. Their pollen grains are rich in lipid oils, causing them to clump into a viscous mass that easily adheres to floral visitors, primarily birds from the families Icteridae, Psittacidae, and Thraupidae [19]. Sucrose concentrations of 7.5% to 10% were favorable for pollen germination [19]. Some authors tested methods for preserving *P. insignis* pollen and found that immersion in petroleum ether for 60 s increased pollen viability [20].

*Platonia insignis* is an allogamous species with a sporophytic self-incompatibility mechanism, primarily breeding through cross-pollination, though some observations have noted fruits from self-fertilization [7]. Fruit formation was not observed after isolating flowers with filo bags.

Few studies have examined the relationship between the phenology of *P. insignis*, its pollinators, and its mating system. While reproduction primarily occurs through seeds, asexual propagation via sprouts emerging from the roots of adult trees is also common, resulting in many *P. insignis* forests consisting of clones [21]. Phenological aspects were observed over three consecutive years, and it was found that flowering lasted from 2 to 4 months, while fruiting lasted almost 9 months. *Platonia insignis* fruits are oblong, angled, recalcitrant, rich in pulp, and typically contain 1 to 5 seeds, with 2 seeds per fruit being the most common. Its germination cycle is lengthy and divided into four stages: (1) tegument breaking (10 to 12 days), (2) primary root growth (12 to 210 days), (3) epicotyl emergence (180 to 900 days), and (4) the emergence of metaphylls [11].

The ability of plants to adapt to environmental changes is crucial in the context of climate change. However, research on *P. insignis* adaptation to climate change is limited. Alvarez et al. [6] characterized the leaf anatomy of *P. insignis* from different secondary forest areas in Pará State (Brazil) during the Amazon summer and winter periods. They found no structural changes but observed phenotypic plasticity in response to varying shading and light intensity levels. Although native to the tropical Amazon, *P. insignis* leaves exhibit xeromorphic anatomical features, such as thick leaves, epicuticular wax, and small stomata, suggesting potential adaptability to climate change.

The scarce literature on the phenology, reproduction, and adaptation mechanisms of *P. insignis* highlights the need for more research to fill knowledge gaps regarding this important Amazonian species.

#### 2.2.2. Diversity, Genetic Resources, and Conservation of *P. insignis*

The center of origin and diversity of *P. insignis* is the Brazilian Amazon region, specifically in the state of Pará [22]. Since the 1990s, the genetic resources of *P. insignis* have faced threats from the timber industry, urban sprawl, and deforestation for cattle breeding in their natural habitats [23]. Combined with the species’ slow germination process, *P. insignis* tree populations are experiencing a significant decline in natural regeneration rates [7]. Given this scenario, the role of gene banks in preserving the valuable genetic material of native *P. insignis* genotypes becomes even more critical.

Germplasm banks play a pivotal role in safeguarding genetic diversity and facilitating the development of high-quality materials by breeders [9]. There are two germplasm banks for *P. insignis* maintained by the Brazilian Agricultural Research Corporation (EMBRAPA). One is located at Tomé-Açu (Pará, Brazil) at Embrapa Amazônia Oriental and holds 118 accessions from Pará and Maranhão States. The other is at Teresina (Piauí, Brazil) at Embrapa Meio Norte, with 77 accessions collected in Piauí and Maranhão States [24].

Initial efforts to characterize these accessions used the physical and chemical parameters of fruits [25]. However, using agronomic and morphological traits is not ideal due to the significant phenotypic variation induced by environmental factors. Additionally, bacuri trees have a long germination period and an extended juvenile phase before flowering and fruiting. Therefore, molecular markers are crucial research tools for accelerating germplasm evaluation and selection in breeding programs aimed at developing improved plant populations [23]. Seven research papers characterizing accessions from these seed banks and *P. insignis* population genotypes were found in the literature. A summary of the main research findings is presented in Table 1.

Souza et al. [23] assessed 72 genotypes from the Embrapa germplasm bank and found high genetic differentiation between sampled populations. The most genetically diverse genotypes were in Barras (Piauí) and Matões (Maranhão), leading to the conclusion that ISSR markers are useful for sampling efforts and germplasm conservation. Pontes et al. [9] used ISSR (Inter-Simple Sequence Repeat) molecular markers to characterize 78 *P. insignis* accessions from the EMBRAPA Amazonia Meio Oeste germplasm bank and assess genetic distance. The authors observed low genetic diversity between sampling localities and minimal genetic differentiation after AMOVA and UPGMA clustering analyses based on 49 ISSR markers. These findings highlight the need for more effective sampling efforts to enrich *P. insignis* germplasm within the seed bank.

Pena et al. [10] conducted a study utilizing ISSR markers to characterize 13 accessions of *P. insignis* from Embrapa Eastern Amazon, originating from various locations in Pará. The authors analyzed 67 polymorphic loci and observed a higher level of polymorphism than Pontes et al. [9] and identified two distinct genetic groups with no differentiation caused by sampling locality. Paraense et al. [27] were the first to develop microsatellite markers for *P. insignis* and noted an elevated polymorphism rate in 59% of the SSR loci amplified, which can assist in genetic breeding efforts that are still lacking for this species.

Nascimento et al. [22] used nuclear and chloroplast microsatellite markers (ncSSR and cpSSR) to investigate the genetic structure and diversity of *P. insignis* populations from various Brazilian states (Table 1). These authors observed greater diversity in Amazonian populations compared to those in the northeastern/Cerrado region, with high genetic structuring among populations, suggesting potential speciation over time. Fixation indexes were negative, indicating an excess of heterozygotes, explained by the reproductive system of this allogamous species. These results suggest that *P. insignis* can adapt to diverse environmental conditions in these biomes and that habitat integrity and pollinator populations are critical for maintaining genetic diversity within *P. insignis* populations.

Fire, urban expansion, and agricultural activities disrupt the natural regeneration of *P. insignis*, leading to reduced populations and threatening its genetic heritage [22]. All authors emphasize the importance of habitat preservation, combating illegal wildlife trade, and germplasm collection to protect against the loss of genetic diversity and the increased genetic structuring of *P. insignis* populations. Although *P. insignis* is native to the Amazon, significant and diverse populations in the Cerrado transition zone and Pre-Amazonic regions of northeastern Brazil suggest that past management practices have contributed to the current genetic diversity scenario. Nascimento et al. [22] also highlighted the role of traditional communities (indigenous and maroon communities) in this management. These communities are currently in danger due to disaggregation, reliance on government aid, and minimal support from specialized agencies [28].

The ideal scenario would involve both utilizing and conserving *P. insignis* genetic resources. However, in situ conservation initiatives remain limited, primarily managed by EMBRAPA (the Brazilian Agricultural Research Corporation) and Brazilian university researchers. This highlights the potential role of Non-Governmental Organizations (NGOs) and the international community in safeguarding the future of *P. insignis*, an invaluable resource of the Amazon.

### 2.3. Production Systems and Management

Carvalho et al. [11] were the only ones to address *P. insignis* germination and seedling development. According to this study, four key morphological events are related to the formation of *P. insignis* seedlings, beginning with radicular protrusion, occurring on average 18 days after sowing. This is followed by the vigorous growth of primary roots, reaching an average length of 180 cm at 210 days. The third event is epicotyl emergence, occurring between 198 and over 960 days, preceding the opening of metaleaves, which happens on average 14 days after epicotyls reach 5 to 7 cm. This article also established that *P. insignis* seeds are sensitive to desiccation, classifying them as recalcitrant.

Conversely, other publications have focused on the interaction of *P. insignis* with its ecosystem and economic use. For example, Menezes et al. [12] examined management techniques used in the cultivation of *P. insignis* among 108 family farmers, finding that the species is primarily exploited through an extractive system, followed by sprout management and seedling planting. The sprouts of *P. insignis* originate from roots and emerge abundantly in secondary forests. Managing these sprouts involves selecting the most vigorous shoots and continuously thinning the others. Although the authors distinguish between extractivism and sprout management, both practices occur in primary or secondary forests where *P. insignis* is already established.

Extractivism involves only clearing to facilitate fruit collection, while sprout management includes rustic practices with a limited scientific basis and technical assistance. In the seedling planting system, Menezes et al. [12] reported the successful formation of seedlings from seeds, the positive response of *P. insignis* to grafting, and the viability of planted orchards, particularly in Tomé-Açú, Pará, Brazil. However, there is a lack of scientific literature providing protocols with high success rates for these practices.

Given the predominance of extractive management, studies published in 2014 and 2020 focused on dendrometric development and the inter- and intra-specific interactions of *P. insignis* in secondary forests. Junior et al. [29] aimed to assess the effect of silvicultural treatments on the diametric growth of *P. insignis*, suggesting that these treatments can double tree growth compared to untreated forests and even quadruple it compared to primary forests. The silvicultural treatments mentioned by these authors included thinning by felling, thinning by girdling, crown pruning, weed clearing, seedling management, and vine cutting. Their results confirmed the hypotheses, showing superior development of *P. insignis* in treated forests compared to untreated ones. Furthermore, da Silva et al. [30] sampled areas under 6, 10, 25, and 100 years of fallow and found that *P. insignis* growth and development are also influenced by the forest regeneration level and vegetative stratum reached by individuals. Both studies corroborate that the economic exploitation of *P. insignis* through extractivism profoundly impacts floral richness and abundance, making *P. insignis* the predominant species in these ecosystems.

Regarding diseases, two fungi have been identified as pathogens for *P. insignis*: Phomopsis, causing rot in mature fruits [31], and Lasiodiplodia pseudotheobromae, causing symptoms of descending drying with resin exudation in the aerial organs of bacuri trees [32]. Although these fungi have been isolated and identified, there are no data on the percentage of fruit or plant loss, nor on the socio-economic impact of these infections. Additionally, research on the physiological consequences for the host plant and effective control methods is lacking.

Research aiming to understand the biological mechanisms of the germination, growth, and development of *P. insignis*, as well as the influence of abiotic factors such as water excess or deficit, light, and temperature on its physiology, is scarce. Such research is necessary and urgent to identify parameters that affect the species and enable its domestication.

### 2.4. Postharvest

Adopting adequate techniques for postharvest fruit conservation is crucial not only to ensure viability for long-distance transportation but also to extend shelf life, thereby contributing to the economic sustainability of fruit production. Fontenele et al. [33] addressed this issue by subjecting *P. insignis* fruits, known as bacuri, coated with polyvinyl chloride (PVC) film, to three different storage temperatures (7, 9, and 11 °C). The fruits were evaluated after 12, 22, and 36 days of storage to determine the preservation potential of these conditions. Their results indicated that temperature variation did not significantly impact the preservation of fruit physicochemical quality. However, over the storage period, there was a reduction in total soluble solids content, total titratable acidity, and total soluble sugars, accompanied by an increase in the pH of the fruits. Despite these changes, it was not possible to determine an optimal period for the ideal consumption or processing of bacuri fruits.

This study emphasizes the need for further research on the postharvest conservation of *P. insignis*. Such research could explore various conservation techniques, such as controlled atmospheres with carbon dioxide (CO_2_) or plant growth regulators, to identify effective fruit preservation practices and estimate the shelf life of bacuri after harvest. Based on this information, robust industrial criteria for the processing, storage, and packaging of bacuri could be established, thereby enhancing financial competitiveness in the marketing of these fruits.

### 2.5. Economic Potential

The technical and scientific development of a species is encouraged by its economic potential, driven by the commercial and social returns it offers. For the *P. insignis* tree, these returns are still largely restricted to its endemic area. Bacuri production is concentrated in the Amazon regions of northern and northeastern Brazil, with the Brazilian Institute of Geography and Statistics (IBGE) identifying the states of Pará, Maranhão, Amazonas, and Tocantins as the only bacuri-producing states. Among these, Pará accounts for 86% of the production, making it the main producer of the fruit in Brazil [1].

*Platonia insignis* has significant potential for its fruit, which is widely industrialized by small businesses to produce pulp, juice, yogurt, jams, jellies, and ice cream. Other parts of the plant are utilized in the wood and agro-industrial sectors [1]. The high production values have a notable impact on the income of these communities, with the price per kilogram of pulp ranging from US$ 2 to US$ 12 [34].

Although *P. insignis* production is concentrated in northern and northeastern Brazil, its inputs are also highlighted and utilized by the pharmaceutical industry to produce cosmetics and as a source of promising bioactive molecules for developing medicines [34]. Table S1 shows the various uses of *P. insignis* bioactive compounds tested against different biological targets.

The economic sector surrounding *P. insignis* still yields a low economic return, primarily because production is predominantly carried out by small farmers engaged in slash-and-burn agriculture, artisanal fishing, and other non-agricultural activities. These farmers often lack organized and structured marketing. As a result, most fruit production and its derivatives are used for the subsistence of small producers. The supply of *P. insignis* still does not meet the national market demand. However, collaborations between researchers, development institutions, and industry could expand the commercialization frontiers of *P. insignis* and its derivatives beyond the national market, boosting the economic sector for this species.

### 2.6. Physicochemical and Nutritional Characteristics of Bacuri Fruits

Bacuri pulp has great acceptability in the northern and northeastern regions due to its unique flavor and aroma, commanding high market value. It is rich in bioactive compounds, vitamins, and minerals, offering important nutritional and nutraceutical properties that make its consumption beneficial (Table 2). Thus, effective conservation methods for the pulp are crucial to ensure product quality and extend shelf life. Bezerra et al. [35] found that bacuri pulps retained their physicochemical characteristics when preserved using combined methods (reducing water activity with different sucrose concentrations [0, 17.4, and 28.6%; *w*/*w*], mild heat treatment, and adding sodium benzoate and sodium metabisulfite) for 120 days at room temperature. These methods resulted in water activity values of 0.985 and no microbiological contamination. Lima da Silva et al. [36] tested the stability of bacuri pulp frozen for 12 months at −20 °C, finding water activity values from 0.987 to 0.994 and soluble solids content ranging from 13.27 to 14.83° Brix. Microbiological analyses showed good stability over the 12 months, with minimal physicochemical changes.

Muniz et al. [37] studied the specific heat, specific mass, thermal diffusivity, and thermal conductivity of *P. insignis* pulp at concentrations between 5 and 20° Brix and temperatures of 25 and 30 °C. They found that specific heat, diffusivity, and electrical conductivity decreased with increasing concentration, while specific mass decreased with rising temperatures across all concentrations, with reductions ranging from 0.15% to 0.92%. These results aid in optimizing industrial processes for heating, cooling, and storage.

Guida et al. [38] investigated the stability and rheological behavior of bacuri pulp. They found that apparent viscosity decreased as the deformation rate increased at all tested concentrations (5, 6, and 10° Brix) and with higher temperatures and dilution. Homogenization increased turbidity and reduced sedimentation, indicating that high agitation rates are necessary to achieve homogeneity and effective processing.

Carvalho et al. [39] studied the physical characteristics of bacuri fruits and the physicochemical properties of pulp adhered to seeds and parthenocarpic segments across different genotypes. They observed that fruits with more seeds had fewer parthenocarpic segments, and higher pulp yields were associated with lower yields of peel and/or seeds. Physicochemical analyses showed significant variations among genotypes. The parthenocarpic segments of genotypes CPATU 105-1, CPATU 116-4, 207-4, and Carananduba exhibited higher total acidity than pulp adhered to seeds, with total soluble solids (TSS) and total acidity values ranging from 9.6 to 25.0 for seed-adhered pulp and 7.0 to 11.4 for parthenocarpic segments. This information helps in selecting genotypes with desirable agronomic traits, such as high pulp yield, for fresh consumption or industrial purposes.

**Table 2:** List of nutrients in bacuri pulp according to the literature.

Nutrient	Content	Location	Authors
Vitamin C	3.96 a 1.76 mg 100 g^−1^	Maranhão	Lima da Silva et al. [36]
Proteins	3.9 ± 0.02%	Maranhão	Mendes et al. [40]
Proteins	10.48 ± 0.30%	Maranhão	Guida et al. [38]
Lipids	0.10 ± 0.01%	Maranhão	Mendes et al. [40]
Lipids	1.49 ± 0.13%	Maranhão	Guida et al. [38]
Total Carbohydrates	13.81 ± 0.01%	Maranhão	Mendes et al. [40]
Total Sugars	6.02 ± 0.31%	Maranhão	Guida et al. [38]
Total Sugars	11.78 ± 0.87%	Maranhão	Lima da Silva et al. [36]
Fiber	11.91 ± 0.28%	Maranhão	Guida et al. [38]

Mendes et al. [40] demonstrated that bacuri pulp could serve as a substrate for probiotic cultures, maintaining viability, growth, and organic acid production. Lactobacilli remained stable after 28 days of refrigerated storage without supplements, and fermentation enhanced the juice’s anti-infective effects against *T. molitor* larvae and enteroaggregative *E. coli*, offering a non-dairy functional product alternative.

Braga et al. [41] prepared bacuri yogurts from UHT whole, semi-skimmed, and skimmed milk, analyzing physicochemical properties and rheology. They found that apparent viscosity was influenced by fat content and temperature variation, affecting texture, which might lead to consumer rejection. However, semi-skimmed and skimmed yogurts, with fat content reductions of 75% and 79.16%, respectively, met health and well-being expectations. These studies highlight bacuri pulp’s potential as a nutrient-rich functional food and its new flavor for various food industry by-products. Bacuri is rich in vitamin C, Ca, K, Mg, Fe, Zn, and Cu and proteins (Table 2) [34].

### 2.7. Chemical Compounds of P. insignis

The fruits of *P. insignis* Mart. are rich in chemical compounds spanning various chemical classes. Studies have shown that *P. insignis* contains components such as saturated and unsaturated fatty acids, prenylated benzophenones, diterpenes, alcohols, xanthones, coumarins, flavonoids, and long-chain hydrocarbons [42]. Below is a description of some of the classes found in *P. insignis* species.

#### 2.7.1. Fatty Acids

Seeds of bacuri (*P. insignis*) contain a high concentration of fatty acids, with a total content of 35% unsaturated acids, of which 20% are trisaturated glycerides (trisaturated 19.5%, monounsaturated desaturated 55%, and diunsaturated monosaturated 25.5%). The main fatty acids found are palmitic (55%) and oleic (32%) acids, with smaller proportions of stearic (6%) and hexadecenoic (3%) acids, and probable traces of myristic, arachidic, and linoleic acids [43]. The high content of fatty acids in *P. insignis* fruits is associated with the presence of oil in the seeds.

#### 2.7.2. Volatile Chemicals

Volatile compounds are widely found in many plant species, and bacuri pulp is known for its distinctive aromas during processing. The authors of [44] identified 12 volatile compounds in bacuri pulp, including linalool, 2-heptanone, (Z)- and (E)-linalool oxides, 2-pentanone, 2-nonanone, cis-hexenyl acetate, methyl dodecanoate and its oxides, α-terpineol, 3,7-dimethyloct-1-en-3,7-ol, and eugenol. The monoterpene linalool was found to be the most abundant component and is responsible for the aromatic character of bacuri pulp [44,45].

Chemical analysis of the aromatic compounds in bacuri fruit demonstrated the presence of several main classes of compounds, such as aliphatic alcohols, aromatic compounds, terpenes, oxygenated terpenes, acids, esters, ketones, and aldehydes. The amount of oxygenated terpenes (linalool, (Z)-linalool furanoxide, (E)-linalool furanoxide, and hotrienol) is higher compared to the other classes [46,47].

#### 2.7.3. Benzophenones Class

*Platonia insignis* is known to contain metabolites such as polyisoprenylated benzophenones, a chemical class rarely found outside this family. The most commonly described polyisoprenylated benzophenone for this species is garcinielliptone FC, which is a tautomeric pair of polycyclic polyprenylated benzophenones. The presence of garcinielliptone FC has been confirmed by several studies [48]. Benzophenones are a class with great structural diversity, sharing a common phenol-carbonyl-phenol skeleton, formed by an A-ring (generally containing 0, 1, or 2 substituents) and a B-ring (prenylated and cyclized). This cyclization produces bi-, tri-, and/or tetra-cyclic ring systems [49].

#### 2.7.4. Bioflavonoids

Bioflavonoids are also representative compounds for the species *P. insignis* and are commonly found in the species, considered chemotaxonomic markers of the Clusiaceae family. The biflavonoids a0072e are grouped into four main groups: GB1, GB-1a, morelloflavone, and amentoflavone [48,50]. Chromatographic peaks, the retention time, the molecular ion, and their fragmentation are detailed in [50]. Morelloflavone and volkensiflavone, two biflavonoids, were isolated from the ethyl acetate fraction of *P. insignis* [51].

#### 2.7.5. Biological Activity of *P. insignis* Compounds

For centuries, humanity has searched for compounds in plants with therapeutic properties for various diseases. Plants are an excellent source of biomolecules for the food, cosmetics, and biotechnology sectors, particularly for drug production. The world’s flora contains numerous plant families that are sources of bioactive compounds with proven biological effects through in vitro and in vivo tests. Among these plant families, Clusiaceae is notable for yielding medicinal compounds.

**Figure 2:**
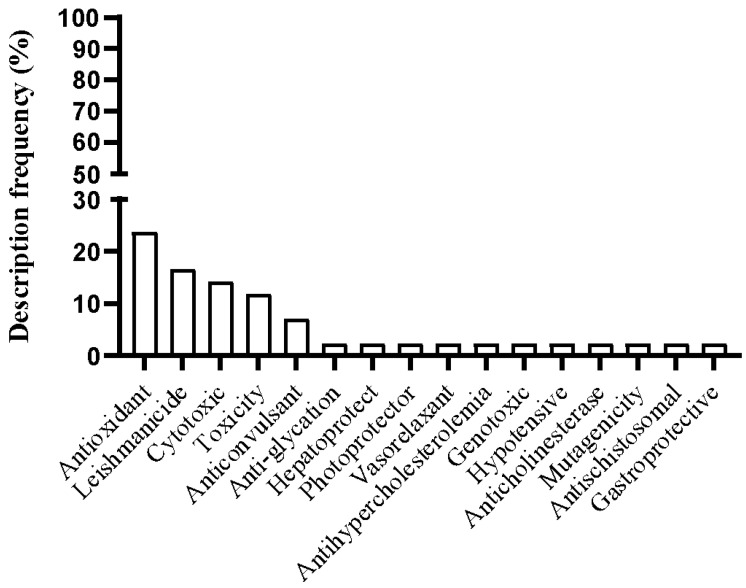
Biological activity of *P. insignis* active compounds described in the literature.

The species *P. insignis*, belonging to the Clusiaceae family, has been described in the literature as possessing various medicinal properties. The frequency of descriptions and the biological actions of *P. insignis* can be seen in Figure 2 and Table S1, respectively. A total of 16 pharmacological properties are described in the literature, with the most frequently cited being antioxidant (23.80%), leishmanicidal (16.66%), cytotoxic (14.28%), toxic (11.90%), and anticonvulsant (7.14%) effects. Other biological activities attributed to *P. insignis* bioactive compounds are described in Figure 2.

The active compounds used to evaluate the biological effects of *P. insignis* come from the seeds, stem, bark, and flowers of the plant, extracted with solvents of different polarities, such as ethanol, hexane, ethyl acetate, and dichloromethane (Table S1). The change in polarity of the extracting solvent contributes to the extraction of molecules that confer the pharmacological action of the species.

Vegetal extracts, fractions, and compounds isolated from *P. insignis* have exhibited antioxidant capacity. Among the tested compounds, the most effective were 2-oleyl-1,3-dipalmitoyl-glycerol (EC50 = 29.92 μg mL^−1^), ethyl acetate extract (EC50 = 33.03 μg mL^−1^), ethanolic extract (EC50 = 59.87 μg mL^−1^), and dichloromethane fraction (IC50 = 90.90 μg mL^−1^) (Table 1) [48,52,53]. Antioxidant activities of plant compounds represent biologically important defenses against reactive oxygen and nitrogen species (ROS and RNS) that can form from any molecule with unpaired electrons. Antioxidant defenses help prevent or reduce oxidative damage to human tissues, potentially preventing the onset of degenerative diseases associated with oxidative stress [53].

In vitro bioassays have extended the leishmanicidal action of *P. insignis* compounds, showing efficacy against promastigote and amastigote forms of the Leishmania genus (Table S1). Bezerra et al. [51] demonstrated high efficiency of extracts and fractions from *P. insignis* flowers, with 50% inhibitory concentration (IC50) values of IC50 = 30.05 μg mL^−1^ (hydroalcoholic extract) and IC50 = 23.05 μg mL^−1^ (ethyl acetate fractions) on promastigotes. A leishmanicidal effect was also observed for the dichloromethane fraction (IC50 = 24.89 µg mL^−1^) obtained from *P. insignis* seeds [52].

Plant compounds have shown promise as methods for treating diseases. As shown in Table S1, *P. insignis* has enormous potential to produce leishmanicidal drugs, which can be combined with synthetic drugs already available on the market to achieve a broad spectrum of drug action. The antileishmanial effects of these compounds are associated with the presence of polyisoprenylated benzophenone (garcinielliptone FC) and triterpenes (lupeol) in the plant material. Data from the literature suggest that lupeol is a biomarker for leishmanicidal activity due to its ability to affect metabolic pathways essential for the parasite’s survival [54].

The cytotoxicity bioassay against tumor cell lines of the garcinielliptone FC compound isolated from *P. insignis* seeds demonstrated an in vitro cytotoxic effect with inhibitory concentrations of 1.4, 3.0, and 3.0 μg mL^−1^ on HL-60 (promyelocytic leukemia), HEP-G2 (human hepatocellular carcinoma), and NCI-H-292 (lung carcinoma) cell lines, respectively [55]. Cytotoxicity assays conducted by the authors of [51] also showed low cytotoxic effects on murine macrophages and sheep erythrocytes for extracts, fractions, and isolates of *P. insignis* using the MTT (3-[4,5-dimethylthiazol-2-yl]-2,5-diphenyltetrazolium bromide) test. The non-cytotoxic activity of garcinielliptone FC was observed in rats treated with dose concentrations of 500, 1000, and 2000 mg kg^−1^, with no signs of toxicity or mortality during the assessment period [56,57]. Cytotoxicity and toxicity bioassays of plant compounds are essential to ensure the safe use of these bioactive compounds, confirming that the chemical compound is selective to the target organism and not harmful to cells. Although many plant molecules are considered safe for medicinal use, inappropriate use can lead to cell poisoning.

### 2.8. Biotechnological Potential of P. insignis

#### 2.8.1. In Vitro Cultivation and Potential of Bacuri In Vitro Banks

Tree domestication is less common compared to agronomic crops due to various obstacles, particularly for *P. insignis*. This species faces an early and slow domestication process, an imminent risk of genetic erosion due to intensive land use for agriculture and livestock, difficulties in seed propagation due to slow germination, a long juvenile period, and genetic self-incompatibility [2,7,12,14]. Tissue culture techniques can help overcome these obstacles, especially for fruit trees from the Amazon biome. Plant tissue culture, or in vitro cultivation, involves growing small parts of plants, such as cells, tissues, or organs, into complete plants [58]. This process includes isolating, disinfecting, and maintaining plant fragments in a specific and ideal culture medium in an aseptic environment for a certain period [59].

Micropropagation through tissue culture is an effective method for rapid, uniform, and sustainable production of woody species. This technique allows clonal multiplication using meristematic or non-meristematic cells or tissues as explants [60]. The two most common types of plant regeneration in micropropagation are somatic embryogenesis and de novo organogenesis, which can occur directly or indirectly, depending on whether an intermediate callus stage is needed [61]. Despite ongoing research on the micropropagation of woody plants, little attention is given to Amazonian fruit trees like *P. insignis*. Therefore, it is crucial to gather information on in vitro cultivation of this species. Proposals for in vitro cultivation protocols for *P. insignis* have shown favorable plant establishment rates.

A crucial factor for successful in vitro cultivation is the decontamination stage, especially when using material from adult individuals due to contaminants [62]. Research has shown that decontaminating leaf and root explants using 0.25% calcium hypochlorite and 1.75% sodium hypochlorite with antifungal solutions (Carboxin + Thiram, Carbendazim, Chlorothalonil + Thiophanate-methyl) for 30 min resulted in total decontamination without causing tissue necrosis [63,64].

The micropropagation technique for *P. insignis* demonstrated genetic variability within the genus Platonia among accessions from Maranhão, Brazil [14]. These authors suggested selecting genotypes based on callogenesis, oxidation percentage, and root and shoot formation according to collection cities, namely, AC.7 (Bacabeira), AC.2 (Bacabeira Santa Luzia), AC.1 (Codó), AC.6 (Codó-Bom Jesus), and AC.8 (Morros), with higher success probabilities for in vitro regeneration protocols. This research provides a foundation for future studies on *P. insignis* in vitro micropropagation in the Amazon and supports investigations related to genetic parameters and selection for in vitro regeneration. Although limited, the data demonstrate that *P. insignis* micropropagation is promising.

#### 2.8.2. Genetic Breeding of *P. insignis*

Bacuri fruit is a favorite in the Amazon region, but the lack of domestication poses a significant challenge to its agro-economic potential. Much of the research on bacuri breeding is conducted by EMBRAPA researchers and published as technical bulletins, leading to a scarcity of published papers in scientific databases and highlighting a knowledge gap in this field.

Genetic breeding experiments for selecting superior genotypes require multiple, periodic evaluations involving large experiments with numerous offspring and the evaluation of many repeatable characteristics. Understanding the correlation of different agronomic traits is also crucial for guiding breeding efforts and making more efficient selections, thereby maximizing genetic gains in each selection cycle [65].

Neto et al. [65] found that bacuri traits such as fruit length, fruit diameter, shell weight, and internal cavity volume are highly repeatable, while traits like the number of seeds, inside fruit weight, and seed percentage are heavily influenced by the environment. These authors also identified significant correlations between phenotypic traits: positive correlations between fruit size traits and pulp weight and negative correlations between total pulp weight and the percentage of shells and seeds. Traits important to the market, such as internal fruit cavity, Brix, and total titratable acidity, showed intermediate repeatability, with high repeatability observed in bacuri fruits from different genotypes in Maranhão, northeastern Brazil [66].

De Brito Souza et al. [67] grouped *P. insignis* into five genetically distinct groups based on more than 30 morphological and reproductive characteristics, indicating a high potential for controlled crossings to generate greater heterosis in hybrids. A significant challenge in improving allogamous plants is that the parental genotype is not fully transmitted to the offspring, primarily involving obtaining viable hybrids to serve as parental genotypes. The study found two genotypes with high heterosis that could be used in *P. insignis* breeding programs. The pulp content, fruit length, and fruit length-to-diameter ratio were the traits that most contributed to genetic diversity and dissimilarity between evaluated genotypes.

Neto et al. [65] analyzed 10 fruits from 13 matrices, while Silva et al. [66] characterized 8 fruits from 6 genotypes, suggesting a minimum of 20 fruits for most characteristics. Lately, Maia et al. [68] conducted a more complex analysis using an incomplete block design, sampling 39 progenies from Maranhão and Piauí, and evaluating a minimum of 10 fruits per matrix (5 to 28 depending on the clone). Using indirect selection simulations, these authors found that selecting traits like fruit weight, diameter, or length could result in positive genetic gains for the other traits. However, selecting genotypes that produce smaller fruits could lead to an 11.4% gain in bacuri pulp content, but this would negatively affect the weight and diameter of the fruit.

The *Platonia insignis* genome has not been fully sequenced, so marker-assisted selection cannot yet be applied to this species, leaving it an unexplored research area. As with any wild species in the domestication process, the literature on *P. insignis* is not unanimous and lacks sufficient data to confidently identify genetic material with high repeatability for desired traits (pulp content and oil in the seed). Therefore, significant efforts are necessary to understand and manage cross-pollination experiments, eliminate inferior genotypes, and stimulate the recombination of superior genotypes [68].

The main obstacles in bacuri enhancement projects are related to its life cycle and physiology, including the late apical emergence of seedlings, a prolonged juvenile phase before flowering, and allogamy with minimal self-pollination. This scenario highlights the importance of bacuri enhancement research, as well as fundamental studies on its mating system and physiological mechanisms, to better understand this promising Amazonian fruit.

## 3. Materials and Methods

This systematic review was conducted according to the Preferred Reporting Items for Systematic Reviews and Meta-Analyses (PRISMA) guidelines [69]. A systematic literature search was conducted in January and February 2023 using the Web of Science (WOS), SCOPUS, Brazilian Agricultural Research Database (BDPA), and PUBMED databases. The search filter used was “title, abstract, and keywords” with the keywords “*Platonia insignis*”; “Bacuri”; “Bacurizeiro”, separated by the boolean operator “or.” “Bacuri” and “Bacurizeiro” are popular names for the species in Brazil. Specifically, in the BDPA database, since it does not contain the “title, abstract, and keywords” filter, the filter “article in the indexed journal” was selected.

**Figure 3:**
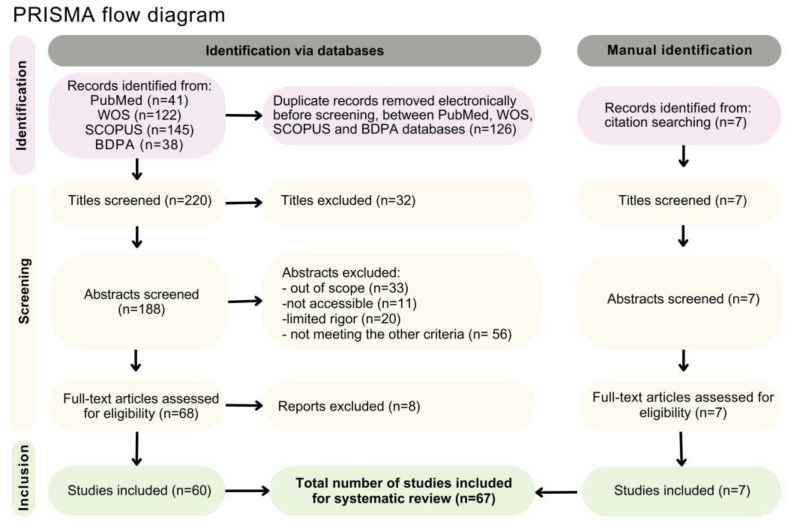
Flowchart of the article selection process in the systematic review.

The number of studies extracted from each database is presented in Figure 3. The bibliographies were exported and merged using the Rayyan web system, which was used for duplicate exclusion and initial screening.

The titles, abstracts, and keywords were read, and the following criteria were observed for the eligibility of the studies: they had to be conducted exclusively with *P. insignis*; they could not be literature reviews; they could not be informative or technical circulars; they could not be book chapters; they could not be papers presented at conferences or events; and they had to be indexed in journals. These filters and criteria were selected to prioritize, primarily in the BDPA, which houses the collection of EMBRAPA’s works, studies subjected to scientific standards and published as peer-reviewed articles, excluding others. This is justified by the fact that, without the application of filters, the search in the BDPA returned 312 records, consisting mainly of technical circulars, informational materials, conference abstracts, and book chapters. This approach aims to meet the objective of the systematic review without disregarding the relevance of the excluded works.

The articles were grouped into related subjects and analyzed to present the results obtained by the authors, the state of the art of each topic, potentials, and possible lines of research for future development.

## 4. Conclusions

*Platonia insignis* is a tree species from the Amazon valued for its timber and processed and fresh fruit pulp, which has nutraceutical properties. However, the species is largely appreciated only within the Amazonian countries, limiting its global recognition.

There is considerable scientific research on the medicinal properties and active ingredients of *P. insignis*, but studies on its economic forestry are lacking. This gap presents a significant obstacle to establishing commercial orchards. Additionally, research focused on understanding the species’ biology and management is limited, highlighting several areas that require further investigation.

Biotechnological techniques are essential for understanding the reproductive biology of *P. insignis*, accelerating epicotyl emergence, seedling establishment, and micropropagation. These methods are crucial for preserving genetic resources from erosion caused by anthropogenic actions. Recent research has focused on plant tissue culture, which could serve as a foundational step toward developing cloning strategies that alleviate pressure on natural populations and directly support domestication and tropical fruit cultivation.

## Figures and Tables

**Table 1:** Summary of the main studies using molecular markers for *P. insignis* germplasm characterization.

Molecular Marker	N° of Accessions	N° of Populations	Location	Mean He/ Genotypes	Authors
ISSR	72	10	Maranhão and Piauí (Brazil)	0.32 to 0.33	Souza et al. [23]
ISSR	30	2	Maranhão (Brazil)	0.33 to 0.38	Santos et al. [26]
ISSR	78	-	Pará (Brazil)	-	Pontes et al. [9]
ISSR	31	-	Pará (Brazil)	-	Pena et al. [10]
SSR	31	-	Pará (Brazil)	0.74 to 0.12	Paraense et al. [27]
Chloroplast SSR	62	7	Amazon, Pará, Maranhão and Piauí (Brazil)	0.47 to 0.66	Nscimento et al. [22]

## Data Availability

All the original contributions presented in the study are included in the article, and further inquiries can be directed to the corresponding author.

## Supplementary Materials

The following supporting information can be downloaded at: https://www.mdpi.com/article/10.3390/plants14060884/s1, Table S1: List of active compounds found in *P. insignis*, extraction methods, chemical components, and doses for different biological activities [51,70,71,72,73,74,75,76,77,78,79,80].
