# Supplementary material for: Platonia insignis: A Systematic Synthesis of Scientific Studies on Its Biology, Ecology, and Potential Applications
Source: Plants (Basel). 2025 Mar 12;14(6):884. doi: 10.3390/plants14060884 (PMC11946368; doi:10.3390/plants14060884)
Supplement: Supplementary file 1 [file plants-14-00884-s001.zip › plants-3477237-supplementary.pdf]

Supplementary information

Platonia insignis: A Systematic Synthesis of Scientific Studies on its Biology, Ecology, and Potential Applications

Kira Figueredo Loiola Alves[1], Aldilene da Silva Lima[2], Priscila Marlys Sá Rivas[1], Irislene Cutrim Albuquerque[1], Jordanya Ferreira Pinheiro[1], Paulo Henrique Aragão Catunda[3], Sérgio Heitor Sousa Felipe[1], Fabrício de Oliveira Reis[1], Diego Silva Batista[4*], Juliane Maciel Henschel[4], Fábio Afonso Mazzei Moura de Assis Figueiredo[1], Antônia Alice Costa Rodrigues[1], Thais Roseli Corrêa[1], Tiago Massi Ferraz[1]

1 Postgraduate Program in Agricultural Sciences, Tissue Culture Laboratory, State University of Maranhão, Av. Lourenço Vieira da Silva - São Cristóvão, São Luís, Maranhão - Brazil, 65055-310;
priscila.sarivas@gmail.com (P.M.S.R.); albuquerqueiris0@gmail.com (I.A.C.); jordanyaf.p@gmail.com (J.F.P.); sergio.h.s.felipe@gmail.com (S.H.S.F.); fareoli@gmail.com (F.d.O.R.); figueiredo.uema@gmail.com (F.A.M.M.d.A.F.); aacrodrigues@outlook.com (A.A.C.R.); thaiscorrea@professor.uema.br (T.R.C.); ferraztm@gmail.com (T.M.F.);

2 Center for Higher Studies of Coelho Neto, State University of Maranhão, Coelho Neto - Maranhão, Brazil; aldilene29@gmail.com (A.d.S.L.);

3 Professional Master's Program in National Network for Management and Regulation of Water Resources, State University of Maranhão, São Luís 65055-310, MA, Brazil; paulocatunda.uema@gmail.com (P.H.A.C.);

4 Postgraduate Program in Agronomy, Federal University of Paraíba, 58397-000, Areia, Paraíba, Brazil
diegoesperanca@gmail.com (D.S.B.); julianemhenschel@gmail.com (J.M.H.)

* Correspondence: diegoesperanca@gmail.com (D.S.B.)

Table S1: List of active compounds of *P. insignis* tested for different biological activities.

Plant part	Extract/ Fraction/ Isolate	Chemical compound	Biological effect	Dose	Authors
Seeds	Hexane extract	Prenylated benzophenone (ɣ-mangostin)	Gastroprotectiv e	50 mg kg^{-1} = 67.40%	[42]
Seeds	Hexane extract	Garcinielliptone FC	Leishmanicidal	IC_{50} = 25.78 mg mL^{-1}	[55]
	Hexane extract	Garcinielliptone FC	Cytotoxic	IC_{50}= 1.4 mg mL^{-1}	
Seeds	Ethyl acetate fraction	1,3,6-Trihydroxy-7-methoxy-2,8-bis	Antioxidant	10 mg kg^{-1} = 1.11	[70]
	Ethyl acetate fraction	1,3,6-Trihydroxy-7-methoxy-2,8-bis	Anticonvulsant	10 mg kg^{-1} = 100%	
Seeds	Hexane extract	Garcinielliptone FC	Cytotoxic	30 µg mL^{-1} = 31.50%	[71]
Seeds shell	Hexane extract	Morelloflavone	Antioxidant	EC_{50}= > 100 µg mL^{-1}	[50]
	Ethyl acetate extract		Antioxidant	EC_{50}= 33.03 µg mL^{-1}	
	Ethyl acetate extract		Anti-glycation	150 µg mL^{-1} = 80%	
Fruit	Bacuri butter	*	Leishmanicidal /promastigote	IC_{50} = 134.77 µg mL^{-1}	[72]
		*	Leishmanicidal /amastigote	IC_{50}= > 800 µg mL^{-1}	
Seeds			Antioxidant	IC_{50}= 141.80 µg mL^{-1}	[52]

	Ethyl acetate fraction	1,3,6-trihydroxy-7-methoxy-2,8-bis (3-methylbut-2-enyl) xanthen-9-one (alpha-mangostin)	Leishmanicidal /promastigote	IC_{50}= 26.20 µg mL^{-1}	
			Toxicity	IC_{50}= 129.0 µg mL^{-1}	
			Antioxidant	IC_{50}= 90.90 µg mL^{-1}	
	Dichloromethane fraction	1,3,5,6-tetrahydroxy-2-(2-methylbut-3-en-2-yl)-7-(3-methylbut-2-enyl)xanthen-9-one (gamma-mangostin)	Leishmanicidal /promastigote	IC_{50}= 2.84 µg mL^{-1}	
			Toxicity	IC_{50}= 24.89 µg mL^{-1}	
			Antioxidant	*	
Fruit	Bacuri butter	Garcinielliptone FC	Hepatoprotective	*	[73]
Seeds	Ethyl acetate fraction	Garcinielliptone FC	Antioxidant	*	[74]
	Dichloromethane fraction		Antioxidant	*	
Branches	Ethanolic extract	Garcinielliptone FC, Morelloflavone, GB-2a	Antioxidant	EC_{50}= 59.87 µg mL^{-1}	[75]
	Hexane fraction			EC_{50}= 120.16 µg mL^{-1}	
	Ethyl acetate fraction			EC_{50}= 99.24 µg mL^{-1}	
	Ethanolic extract		Photoprotector	100 mg mL^{-1} = 15 FPS	
	Hexane fraction			100 mg mL^{-1} = ~10 FPS	

	Ethyl acetate fraction			100 mg mL^{-1} = ~27 FPS	
Fruit seeds	Garcinielliptone FC	Garcinielliptone FC	Vasorelaxant	PHE, 10^{-5} mol L^{-1}	[76]
Seeds	Bacuri seed butter	*	Anti-hypercholesterolemia	25 mg kg^{-1} day^{-1} = ~ 50 mg dL^{-1} (HDL)	[73]
Seeds	Hexane extract	Garcinielliptone FC	Toxicity	500 mg kg^{-1} = 0%	[77]
Seeds	Garcinielliptone FC	Garcinielliptone FC	Toxicity/genotoxic	50 mg kg^{-1} = 0%	[78]
	Ethanol extract	*	Hypotensive	50 mg kg^{-1} = -17.6 mmHg	[79]
Fruit shells	Ethyl acetate fraction	*		50 mg kg^{-1} =-30.0 mmHg	
	hydroalcoholic extract		Antileishmanial	IC_{50} =30.05 µg mL^{-1}	
	Ethyl acetate fractions			IC_{50} = 23.05 µg mL^{-1}	
	Morelloflavone/volkensiflavone mixture	Morelloflavone and volkensiflavone		IC_{50} = 45.71 µg mL^{-1}	[80]
	Hydroalcoholic extract		Cytotoxicity	CC_{50} = 81.78 µg mL^{-1}	
Flowers	Ethyl acetate fractions			CC_{50} = 159.67 µg mL^{-1}	

	Morelloflavone/volkensiflavone mixture			CC_{50} = 134.28 μg mL^{-1}	
Seeds	2-oleyl-1,3-dipalmitoyl-glycerol	2-oleyl-1,3-dipalmitoyl-glycerol	Anticholinesterase	1.0 mg mL^{-1} = 67.34%	[81]
Seeds	2-oleyl-1,3-dipalmitoyl-glycerol	2-oleyl-1,3-dipalmitoyl-glycerol	Toxicity	LD_{50} = < 1200 μg mL^{-1}	[53]
			Cytotoxicity	0.2 mg mL^{-1} = 29.89%	
			Mutagenicity	0.2 mg mL^{-1} = 6.90%	
			Antioxidant	EC_{50} = 29.92 μg mL^{-1}	
Seeds	Dichloromethane fraction		Antioxidant	400 mg kg^{-1} = 94%	[70]
			Anticonvulsant	2 mg kg^{-1} = 100%	
Stem Barks	Hydroalcoholic extract	*	Leishmanicidal	IC_{50} = 174.24 μg mL^{-1}	[54]
	Hexane extract	Lupeol		IC_{50} = 45.23 μg mL^{-1}	
	Hydroalcoholic extract	*	Cytotoxic	CC_{50} = 341.95 μg mL^{-1}	
	Hexane extract	Lupeol		CC_{50} = 71.65 μg mL^{-1}	
Seeds	Hexane extract	*	Cytotoxic	CC_{50} = 90.03 g mL^{-1}	[57]
Seeds	Garcinielliptone FC	Garcinielliptone FC	Antischistosomal	50 μM = 100%	[75]
Seeds	Garcinielliptone FC	Garcinielliptone FC	Anticonvulsant	75 mg kg^{-1} = 90.1%	[77]

* Data not found in the literature

References

42. Lima Nascimento, J.; Coelho, A.G.; Oliveira Barros, Y.S.; Sousa Oliveira, I.; Vieira da Silva, F.; Custódio Viana, A.F.S.; Araújo, B.Q.; dos Santos Rocha, M.; das Chagas Pereira de Andrade, F.; de Oliveira Barbosa, C.; et al. Production and Characterization of a β-Cyclodextrin Inclusion Complex with Platonia Insignis Seed Extract as a Proposal for a Gastroprotective System. *Applied Sciences* 2022, *13*, 58, doi:10.3390/app13010058.

50. Ribeiro, D.C.; Russo, H.M.; Fraige, K.; Zeraik, M.L.; Nogueira, C.R.; da Silva, P.B.; Codo, A.C.; Calixto, G.M.F.; de Medeiros, A.I.; Chorilli, M.; et al. Bioactive Bioflavonoids from Platonia Insignis (Bacuri) Residues as Added Value Compounds. *J Braz Chem Soc* 2021, *32*, 786–799, doi:10.21577/0103-5053.20200230.

52. Costa Júnior, J.S.; Ferraz, A.B.F.; Sousa, T.O.; Silva, R.A.C.; De Lima, S.G.; Feitosa, C.M.; Citó, A.M.G.L.; Melo Cavalcante, A.A.C.; Freitas, R.M.; Moura Sperotto, A.R.; et al. Investigation of Biological Activities of Dichloromethane and Ethyl Acetate Fractions of Platonia Insignis Mart. Seed. *Basic Clin Pharmacol Toxicol* 2013, *112*, 34–41, doi:10.1111/j.1742-7843.2012.00924.x.

53. do Nascimento Cavalcante, A.; Lima, L.K.F.; Araújo, C.M.; da Silva Santos, F.P.; do Nascimento, M.O.; de Castro e Sousa, J.M.; Rai, M.; Feitosa, C.M. Toxicity, Cytotoxicity, Mutagenicity and in Vitro Antioxidant Models of 2-Oleyl-1,3-Dipalmitoyl-Glycerol Isolated from the Hexane Extract of Platonia Insignis MART Seeds. *Toxicol Rep* 2020, *7*, 209–216, doi:10.1016/j.toxrep.2020.01.014.

54. Souza, A.C.; Alves, M.M. de M.; Brito, L.M.; Oliveira, L.G.D.C.; Sobrinho-Júnior, E.P.C.; Costa, I.C.G.; Freitas, S.D.L.; Rodrigues, K.A. da F.; Chaves, M.H.; Arcanjo, D.D.R.; et al. *Platonia Insignis* Mart., a Brazilian Amazonian Plant: The Stem Barks Extract and Its Main Constituent Lupeol Exert Antileishmanial Effects Involving Macrophages Activation. *Evidence-Based Complementary and Alternative Medicine* 2017, *2017*, doi:10.1155/2017/3126458.

55. Júnior, J.S.C.; De Almeida, A.A.C.; De Barros Falcão Ferraz, A.; Rossatto, R.R.; Silva, T.G.; Silva, P.B.N.; Militão, G.C.G.; Citó, A.M.D.G.L.; Santana, L.C.L.R.; De Amorim, F.A.; et al. Cytotoxic and Leishmanicidal Properties of Garcinielliptone FC, a Prenylated Benzophenone from Platonia Insignis. *Nat Prod Res* 2013, *27*, 470–474, doi:10.1080/14786419.2012.695363.

57. Lustosa, A.K.M.F.; Arcanjo, D.D.R.; Ribeiro, R.G.; Rodrigues, K.A.F.; Passos, F.F.B.; Piauilino, C.A.; Silva-Filho, J.C.; Araújo, B.Q.; Lima-Neto, J.S.; Costa-Júnior, J.S.; et al. Immunomodulatory and Toxicological Evaluation of the Fruit Seeds from Platonia Insignis, a Native Species from Brazilian Amazon Rainforest. *Revista Brasileira de Farmacognosia* 2016, *26*, 77–82, doi:10.1016/j.bjp.2015.05.014.

70. Da Costa, J.S.; De Almeida, A.A.C.; Tomé, A. da R.; Citó, A.M. das G.L.; Saffi, J.; De Freitas, R.M. Evaluation of Possible Antioxidant and Anticonvulsant Effects of the

Ethyl Acetate Fraction from Platonia Insignis Mart. (Bacuri) on Epilepsy Models. *Epilepsy and Behavior* 2011, *22*, 678–684, doi:10.1016/j.yebeh.2011.09.021.

71. da Silva Prado, L.; da Silva, J.; Garcia, A.L.H.; Boaretto, F.B.M.; Grivicich, I.; Conter, L.U.; de Oliveira Salvi, A.; Reginatto, F.H.; Vencato, S.B.; de Barros Falcão Ferraz, A.; et al. Evaluation of DNA Damage in HepG2 Cells and Mutagenicity of Garcinielliptone FC, A Bioactive Benzophenone. *Basic Clin Pharmacol Toxicol* 2017, *120*, 621–627, doi:10.1111/bcpt.12753.

72. Coêlho, E. de S.; Lopes, G.L.N.; Pinheiro, I.M.; Holanda, J.N.P. de; Alves, M.M. de M.; Carvalho Nogueira, N.; Carvalho, F.A. de A.; Carvalho, A.L.M. Emulgel Based on Amphotericin B and Bacuri Butter (Platonia Insignis Mart.) for the Treatment of Cutaneous Leishmaniasis: Characterization and in Vitro Assays. *Drug Dev Ind Pharm* 2018, *44*, 1713–1723, doi:10.1080/03639045.2018.1492610.

73. Lindoso, J.V.D.S.; Alencar, S.R.; Dos Santos, A.A.; Mello Neto, R.S.; Mendes, A.V. da S.; Furtado, M.M.; da Silva, M.G.; da Silva Brito, A.K.; Batista, E.K.F.; Baêta, S. de A.F.; et al. Effects of "Bacuri" Seed Butter (Platonia Insignis Mart.), a Brazilian Amazon Fruit, on Oxidative Stress and Diabetes Mellitus-Related Parameters in STZ-Diabetic Rats. *Biology (Basel)* 2022, *11*, doi:10.3390/biology11040562.

74. Costa Júnior, J.S. da; Ferraz, A. de B.F.; Feitosa, C.M.; Citó, A.M. das G.L.; Saffi, J.; Freitas, R.M. de Evaluation of Effects of Dichloromethane Fraction from Platonia Insignis on Pilocarpine-Induced Seizures. *Revista Brasileira de Farmacognosia* 2011, *21*, 1104–1110, doi:10.1590/S0102-695X2011005000163.

75. Silva, A.P.; Silva, M.P.; Oliveira, C.G.; Monteiro, D.C.; Pinto, P.L.; Mendonça, R.Z.; Costa Júnior, J.S.; Freitas, R.M.; de Moraes, J. Garcinielliptone FC: Antiparasitic Activity without Cytotoxicity to Mammalian Cells. *Toxicology in Vitro* 2015, *29*, 681–687, doi:10.1016/j.tiv.2014.12.014.

76. Arcanjo, D.D.R.; Costa-Júnior, J.S. da; Moura, L.H.P.; Ferraz, A.B.F.; Rossatto, R.R.; David, J.M.; Quintans-Júnior, L.J.; Oliveira, R. de C.M.; Citó, A.M. das G.L.; Oliveira, A.P. de Garcinielliptone FC, a Polyisoprenylated Benzophenone from *Platonia Insignis* Mart., Promotes Vasorelaxant Effect on Rat Mesenteric Artery. *Nat Prod Res* 2014, *28*, 923–927, doi:10.1080/14786419.2014.889136.

77. Ana, A.P.; Lopes, J.S.L.; De S. Vieira, P.; E.a. Pinheiro, E.; Mirna, M.L.; José, J.C.; Da Costa Júnior, J.S.; David, J.M.; De Freitas, R.M. Behavioral and Neurochemical Studies in Mice Pretreated with Garcinielliptone FC in Pilocarpine-Induced Seizures. *Pharmacol Biochem Behav* 2014, *124*, 305–310, doi:10.1016/j.pbb.2014.05.021.

78. Coelho, V.R.; Prado, L.S.; Rossato, R.R.; Ferraz, A.B.F.; Vieira, C.G.; de Souza, L.P.; Pfluger, P.; Regner, G.G.; Valle, M.T.C.; Leal, M.B.; et al. A 28-day Sub-acute Genotoxic and Behavioural Assessment of Garcinielliptone FC. *Basic Clin Pharmacol Toxicol* 2018, *123*, 207–212, doi:10.1111/bcpt.13010.

79. Mendes, M.B.; Da Silva-Filho, J.C.; Sabino, C.K.B.; Arcanjo, D.D.R.; Sousa, C.M.M.; Costa, I.C.G.; Chaves, M.H.; Oliveira, R.D.C.M.; Oliveira, A.P. Pharmacological Evidence of A2-Adrenergic Receptors in the Hypotensive Effect of Platonia Insignis Mart. *J Med Food* 2014, *17*, 1079–1085, doi:10.1089/jmf.2013.0151.

80. Bezerra, É.A.; Alves, M.M. de M.; Lima, S.K.R.; Pinheiro, E.E.A.; Amorim, L.V.; Lima Neto, J. de S.; Carvalho, F.A. de A.; Citó, A.M. das G.L.; Arcanjo, D.D.R. Biflavones from Platonia Insignis Mart. Flowers Promote In Vitro Antileishmanial and Immunomodulatory Effects against Internalized Amastigote Forms of Leishmania Amazonensis. *Pathogens* 2021, *10*, 1166, doi:10.3390/pathogens10091166.

81. do Nascimento Cavalcante, A.; Feitosa, C.M.; da Silva Santos, F.P.; de Sousa, A.P.R.; dos Santos Sousa, R.; de Souza, A.A.; Pinto, B.F.; Araújo, C.M.; Rashed, K. Elaboration and Characterization of the Inclusion Complex between β-Cyclodextrin and the Anticholinesterase 2-Oleyl-1,3-Dipalmitoyl-Glycerol Extracted from the Seeds of Platonia Insignis MART. *J Mol Struct* 2019, *1177*, 286–301, doi:10.1016/j.molstruc.2018.09.067.
